# An Incidental Discovery of Dandy-Walker Malformation on MRI in a 76-Year-Old Male Patient: A Case Report

**DOI:** 10.7759/cureus.84423

**Published:** 2025-05-19

**Authors:** Maira Ahmad, Hira Tahir, Salma Salman

**Affiliations:** 1 General Medicine, Dow University of Health Sciences, Dow International Medical College, Karachi, PAK

**Keywords:** adult, aged, ataxia, cerebellum, dandy-walker syndrome, incidental findings, magnetic resonance imaging, male

## Abstract

Dandy-Walker malformation is the most prevalent posterior fossa anomaly. Dandy-Walker variant, or Dandy-Walker complex, are terms devised to contrast those conditions that do not meet the standard of Dandy-Walker malformation. Dandy-Walker malformation is usually a congenital diagnosis. Our case calls attention to an extraordinary presentation of Dandy-Walker malformation in adulthood.

A 76-year-old male patient initially presented to the Emergency Department at Dow University Hospital at Ojha Campus on the grounds of suspicion of a respiratory infection. COVID-19 was suspected; however, polymerase chain reaction (PCR) testing yielded a negative result. The patient later developed an erythematous and swollen left arm, which pointed towards a diagnosis of cellulitis, and the patient was managed accordingly. After being moved to the General Medicine Ward, our team noted that the patient was bed-bound and conducted a neurological exam, in which he presented with incoordination, less brisk and pendular deep tendon reflexes, and hypotonia in lower limbs, all suggestive of cerebellar involvement. The patient was also lethargic and was in a state of confusion, suggesting an altered mental status. Subsequent labs were completed, and bacterial pneumonia was confirmed on pleural fluid analysis. A CT scan of the head and an MRI scan of the brain were also ordered to investigate the reason for neurological involvement. The imaging displayed hallmarks signifying Dandy-Walker malformation, such as enlargement of the fourth ventricle, hypoplastic vermis, and posterior fossa anomalies.

## Introduction

Dandy-Walker malformation, an uncommon autosomal dominant condition, is denoted by cerebellar vermis hypoplasia, hydrocephalus, and a posterior fossa cyst with a dilated fourth ventricle [1]. It is recognized as the most common cerebellar malformation, occurring in 1 out of every 25,000-30,000 live births, affecting females more than males [2]. Dandy-Walker malformation is poorly understood, with insufficient current data published to determine its significance in developing countries such as Pakistan [3].

This syndrome is typically diagnosed prenatally through neuroradiology or in infancy with clinical findings of an increased head circumference [2]. In infancy, this condition can contribute to motor and cognitive abnormalities, hypotonia, microcephaly, and cleft palate, with signs like nausea, irritability, convulsions, and muscle incoordination presenting early in life [1]. Two-thirds of patients have accompanying central nervous system anomalies, including abnormal brainstem development and agenesis of the corpus callosum [2]. One-third have multi-organ system abnormalities, consisting of cleft lip and palate, and cardiac and urinary issues [4]. 

Adult presentation is rare and is usually found on CT imaging done for patients presenting with head injuries [5]. Older patients may even present with no symptoms and have normal results on neurological examinations [2]. Typical presentations include signs like developmental disabilities, inability to maintain the head upright, seizures, and spastic muscles [2,6]. While the presence of these signs may allow for consideration of Dandy-Walker malformation as the cause, the condition cannot be diagnosed clinically. Instead, the diagnosis is exclusively based on imaging modalities, such as CT and MRI, with MRI of the brain being the best diagnostic choice [2]. However, these imaging modalities are considered variably expensive for the general population in developing countries like Pakistan, with private sectors having more accessibility to these machines, which could account for undiagnosed cases [7].

Dandy-Walker may also present in Mendelian disorders, such as Walker-Warburg syndrome, in which it exists with other conditions leading to multi-system damage [6]. These conditions commonly include macrocephaly and hydrocephalus, holoprosencephaly, and dysgenesis of the corpus callosum. However, it can also be associated with conditions outside of the central nervous system, like cleft palate and cardiac abnormalities [8]. Dandy-Walker can cause problems in patients early on in their lives or later in adulthood [9]. The co-occurrence of Dandy-Walker with these conditions can lead to different presentations within patients, allowing for cases of misdiagnosis, as well as undiagnosed cases of death possibly due to the syndrome.

Most importantly, Dandy-Walker can appear with symptoms or remain inconspicuous, as is present in our patient. Pediatric patients presenting with Dandy-Walker exhibit more detectable symptoms, such as developmental disability, hypotonia, and ataxia [6], which can help with early diagnosis. However, adult case presentations regarding the conditions presenting with Dandy-Walker are rare and more extreme. Some of these cases remain undiagnosed or are diagnosed in old age, and patients are found to have Dandy-Walker after their expiration or late in life [5,10,11]. In these cases, Dandy-Walker can present with severe conditions that are either associated with it or can possibly be caused by it [12,13]. Overall, it is crucial to note that Dandy-Walker malformation is sporadic in adults, especially males [2]. Our 76-year-old male case highlights the variable and asymptomatic presentation in adults, contributing knowledge to the current limited understanding of Dandy-Walker malformation.

## Case presentation

A 76-year-old male patient presented to the Emergency Department at Dow University Hospital with shortness of breath, cough, and fever, and was bed-bound due to fatigue and weakness, with no other respiratory symptoms. Besides fatigue, the patient had no arthralgia, joint pain, or swelling. The patient did not present with syncope, palpitations, orthopnea, or peripheral edema. The patient had no specific gastrointestinal findings, such as vomiting, nausea, appetite changes, jaundice, or any bowel irregularities. He had no urinary issues, such as dysuria or flank pain, and urine color and amount were normal. There were no other remarkable signs or symptoms. 

An initial impression of COVID-19 was made, and the patient was moved to the COVID-19 high-dependency unit, where a polymerase chain reaction (PCR) test was taken and was negative. After COVID-19 was ruled out, the patient was assumed to have pneumonia. However, he was transferred to the Intensive Care Unit (ICU) due to developing redness and swelling of his left arm at the time, upon which the diagnosis of cellulitis was made, and the patient was treated. During his stay in the ICU, the patient's shortness of breath, cough, and fever worsened, and he was shifted to the General Medicine Ward. After presenting to the General Medicine Ward, our medical team proceeded to do imaging, labs, and basic examinations to evaluate the patient, and suspected pneumonia to be the cause of the patient's worsening condition. Daily labs were done to monitor the patient from May 17, 2021, to May 26, 2021. Table 1 summarizes the patient's hematology lab findings.

**Table 1:** Complete blood count results

Test	May 17, 2021	May 18, 2021	May 19, 2021	May 21, 2021	May 24, 2021	May 25, 2021	May 26, 2021	Reference Range
Hemoglobin (g/L)	14.9	17	17	16	17.7	16.5	18.2	13-17
RBC Count (x 10^12^/L)	5.6	6.5	6.5	6.1	6.7	6.4	7.0	4.5-5.5
Hematocrit (%)	48	56	56	54	57	54	60	40-50
Mean Corpuscular Volume (fL)	87	88	86	89	84	85	84	80-100
Mean Corpuscular Height (pg)	27	27	27	26	26	26	26	27-32
Mean Corpuscular Hemoglobin Concentration (gm/dL)	31	31	30	29	31	30	30	31.5-34.5
White Blood Cells (x 10^9^/L)	21.3	28	23	29	36.2	30.2	42.5	4.0-10.0
Platelets (x 10^9^/L)	689	251	836	645	431	370	392	150-400
Neutrophils (%)	90	95	94	95	94	95	94	40-75
Lymphocytes (%)	3	1	1	1	1	1	0	20-45
Monocytes (%)	7	4	4	4	5	4	5	2-10
Eosinophils (%)	0	0	1	0	0	0	0	1-6
Basophils (%)	0	0	0	0	0	0	1	0-1

Upon admission to the General Medical Ward, the patient's history was taken by his nephew and his wife, with whom he lived. The patient had a short history of cough, shortness of breath, and fever for four to five days. He had fatigue and weakness for two to three years, and during this time, he had repeated lower respiratory tract infections. He did not have any surgeries in the past, no allergies or comorbidities, and no significant family history. The patient was, overall, in a bed-bound state, which raised concern for our medical team as to what had caused this state. Thus, the team conducted a neurological and cerebellar examination to check for the extent of neurological damage and to assess which parts of the nervous system may have been affected. Because the patient was weak and bedridden, examinations were limited in areas of gait assessment. However, an upper and lower limb motor exam was performed, for which the patient demonstrated a grade of 3 out of 5 for power, as he could move both upper and lower limbs against gravity, but with difficulty. Reflexes were also checked and were found to be less brisk, and deep tendon reflexes were pendular. The patient was found to have weak muscle tone in both upper and lower limbs, with a grade of 1+ for hypotonia. Moreover, the patient was not properly coordinating, so the cranial nerve examination was very limited, and the sensory examination could not be performed. For the cranial nerve examination, only the vagus and glossopharyngeal nerves were checked, for which the patient had weak gag and palatal reflexes. The patient was also assessed using the Glasgow Coma Scale to assess his level of consciousness and received a score of 11 out of 15. To clarify, the patient was able to open his eyes on verbal commands, giving a score of 3 for 'eye response'; he was able to localize pain, giving a motor response of 5; and he was using random or inappropriate words unrelated to the present conversation at hand, giving a verbal response of 3. Moreover, the patient was lethargic and disoriented.

A limited cerebellar examination was also performed. Assessment of gait, proprioception, nystagmus, dysdiadochokinesia (rapid and alternating movements), rebound test, and heel-to-shin test were not done due to the patient's state of health at the time, as mentioned before. However, the patient had a weak intentional tremor and incoordination on the finger-to-nose test and demonstrated slurred speech. The patient's nephew explained that the patient had been experiencing weakness and fatigue for two to three years, but they attributed it to the process of normal aging. During this time, the patient had been to one clinic for an acute illness, but this state of severe weakness was not investigated. Overall, this was his first visit to our hospital, where, during his admission for pneumonia, he was investigated and diagnosed with neurological deficits for the first time. 

The patient's worsening condition led him into a state of sepsis during his stay in the General Medicine Ward from May 17, 2021, to May 26, 2021. At the time, the patient was also found to have an electrolyte imbalance and had been experiencing lethargy, disorientation, and confusion since presenting to the General Medicine Ward. The patient was constantly monitored for electrolyte levels while also being managed with fluids and electrolytes to correct the imbalance. Our team suspected that the electrolyte imbalance was actually the cause of the patient's current state of lethargy, disorientation, and confusion. The daily biochemistry labs done from May 17, 2021, to May 26, 2021, are summarized in Table 2. 

**Table 2:** Biochemistry labs results

Test	May 17, 2021	May 18, 2021	May 19, 2021	May 21, 2021	May 24, 2021	May 25, 2021	May 26, 2021	Reference Range
Urea (mg/dL)	53.5	55.64	64.2	53.5	74.9	72.76	74.9	17-49
Creatinine (mg/dL)	0.83	0.72	0.72	0.60	0.51	0.45	0.65	0.9-1.3
Electrolytes								
Sodium (mEq/L)	142	146	152	152	140	141	141	136-146
Potassium (mEq/L)	5.5	4.8	4.3	4.2	4.5	4.4	5.1	3.5-5.1
Chloride (mEq/L)	104	105	111	109	106	105	105	98-107
Bicarbonate (mEq/L)	19.1	24.4	20.6	28.0	24.5	22.6	18.6	23-29
Magnesium (mg/dL)	2.45	2.43	2.63	2.50	-	2.17	2.21	1.6-2.6
Phosphorous (mg/dL)	5.40	3.60	2.60	3.90	2.40	2.30	2.70	2.5-4.5
Calcium (mg/dL)	8.00	8.70	9.10	8.80	7.60	7.30	7.80	8.6-10.2

After electrolytes and fluids came into the normal range, the patient was still in the same state and still presented with cerebellar signs. Thus, the team decided to have a CT of the head done, which was scheduled for May 21, 2021. The CT scan findings concluded that all ventricles appeared to be enlarged and dilated, and were disproportionate to the atrophic changes in the adjacent brain parenchyma. This detail prompted the team to request an MRI of the head for further investigation. 

**Figure 1:**
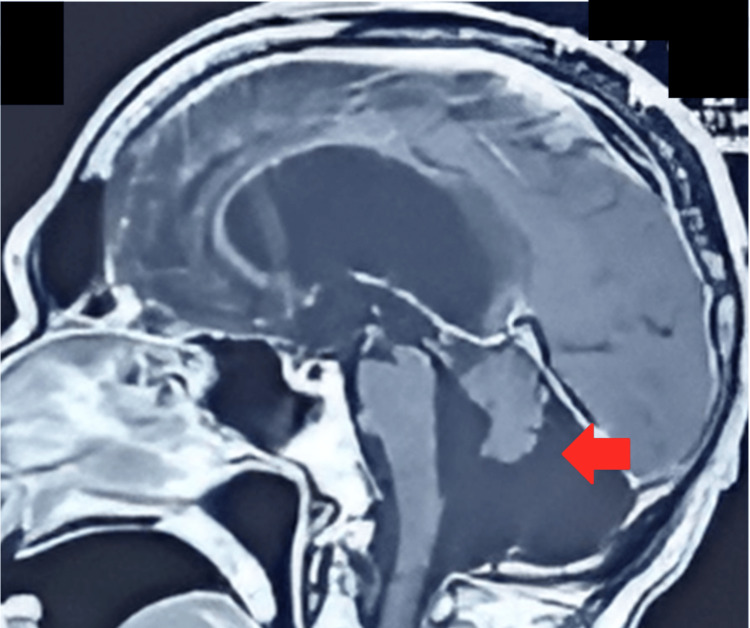
Brain MRI without contrast sagittal view showing a hypoplastic cerebellum and a CSF signal intensity area (arrow) in the posterior fossa communicating with the enlarged fourth ventricle anteriorly

**Figure 2:**
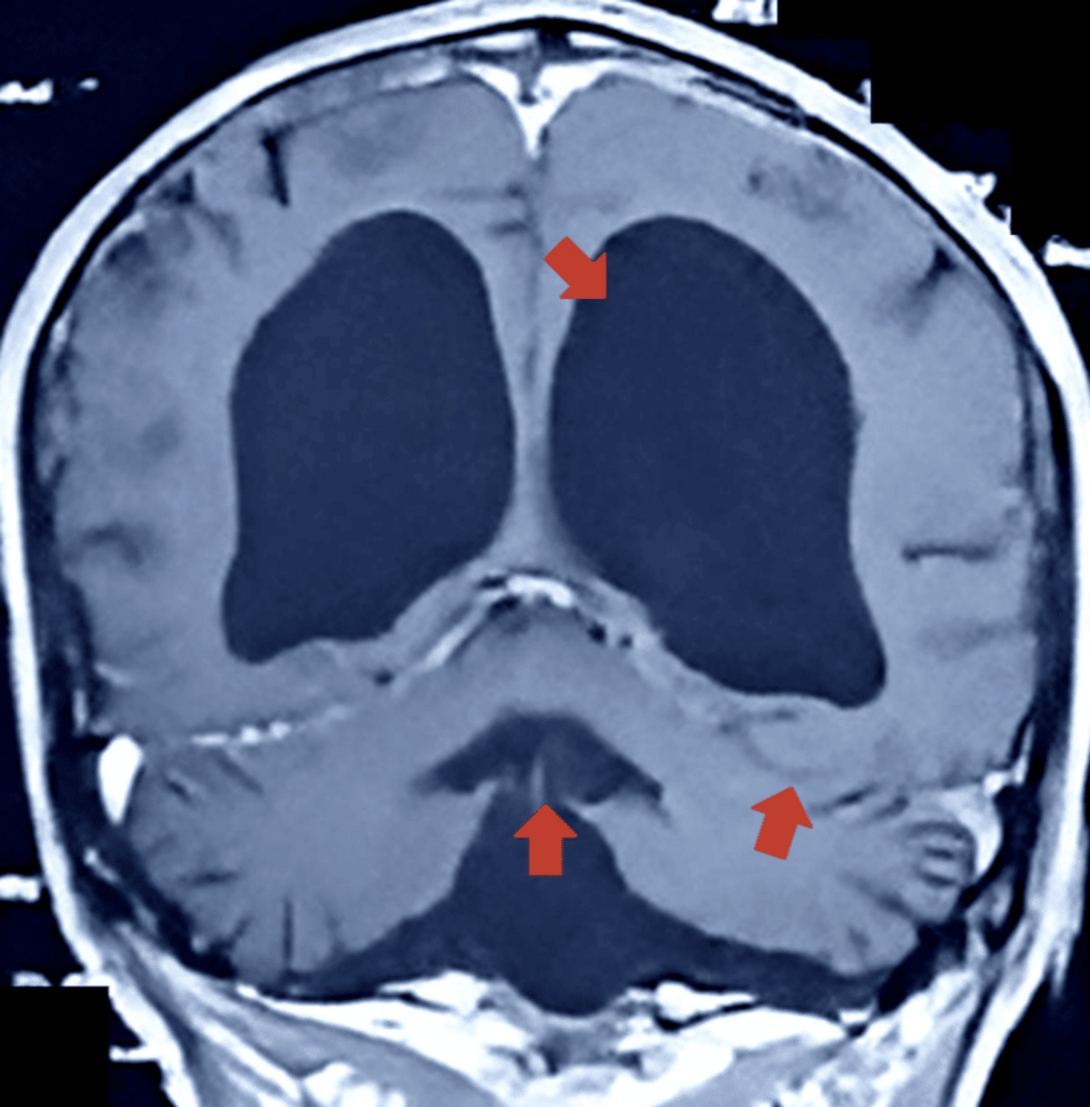
Brain MRI without contrast coronal view showing dilated lateral ventricles, and a hypoplastic vermis, and slight bulging of the tentorium cerebelli (arrows) posteriorly

**Figure 3:**
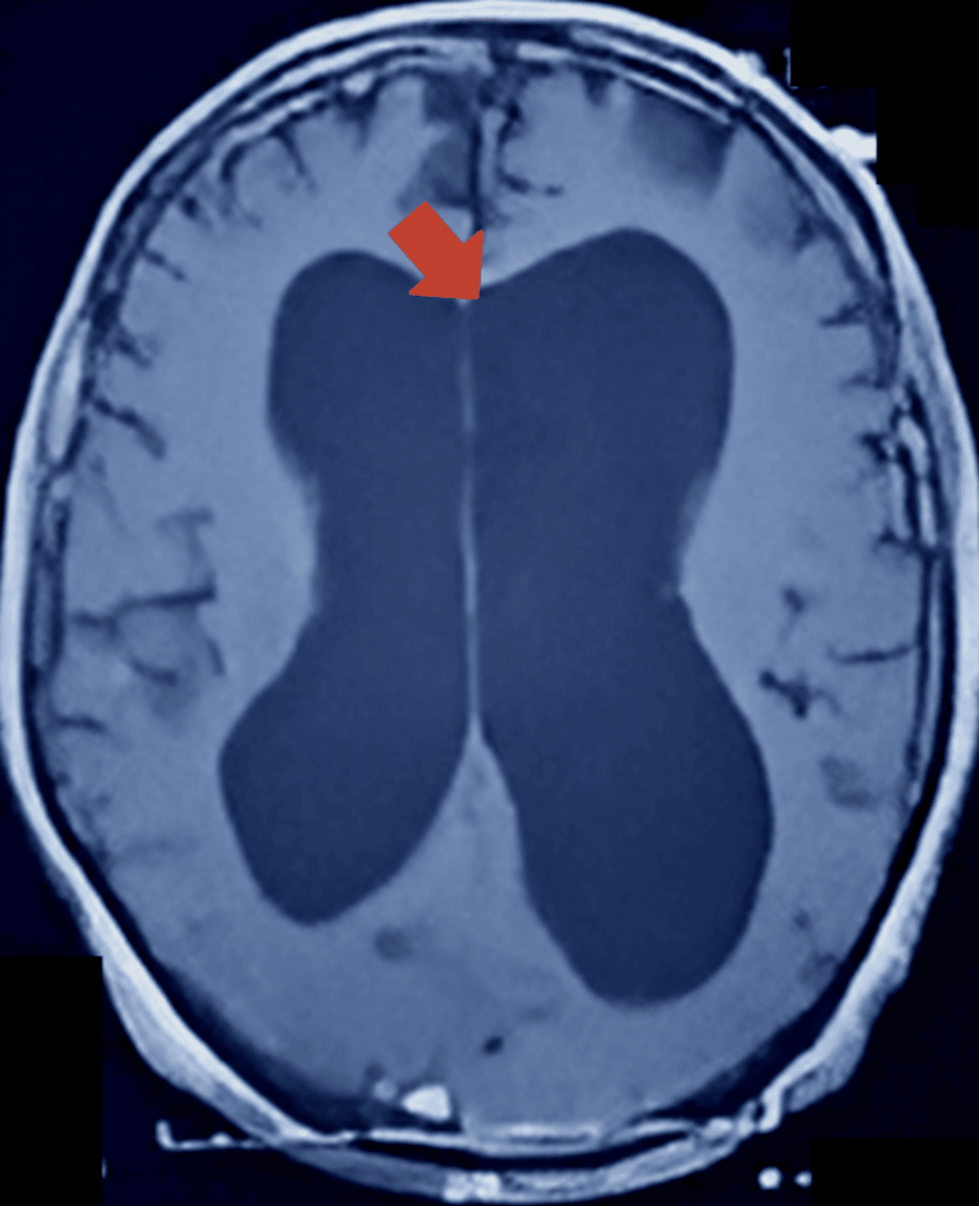
Brain MRI without contrast axial view showing dilated lateral ventricles (arrow)

On May 25, 2021, an MRI of the head/brain with and without contrast revealed a large extra-axial CSF signal intensity area in the posterior fossa, communicating with the fourth ventricle anteriorly (Figure 1). The vermis was hypoplastic, with slight bulging of the tentorium cerebelli. Posteriorly, the bulge was produced over the inner table (Figure 2), with the lateral ventricles seen to be largely dilated (Figure 3).

Based on the findings above, our patient was concluded to have radiological findings closely resembling those found in Dandy-Walker malformation. Differentials included Mega Cisterna Magna and Dandy-Walker variants. The neurology team at Dow was consulted, and nursing care and physical therapy were offered thereafter and started. These options were considered to increase the quality of life for the patient after discharge and to improve the patient's overall mobility. Upon discussion, the neurology team did not advise ventriculoperitoneal shunting to drain excess cerebrospinal fluid and address ventricular dilation. This was due to considering factors like the patient's advanced age, the current state of health due to infection, and the decreased benefits from such a major procedure, which ultimately made ventriculoperitoneal shunting an inviable option for our patient.

On May 26th, 2021, a chest X-ray was ordered for the patient and showed blunting of the costophrenic angles and fluid in the lungs, confirming that the patient had developed a pleural effusion. Our team also ordered a pleural fluid analysis and cultures to determine the exact etiology. The pleural fluid analysis confirmed bacterial pneumonia, with results summarized in Table 3.

**Table 3:** Pleural fluid analysis μL: microliter; RBC/HPF: red blood cells/high power field; g/dL: grams per deciliter

Pleural Fluid Diagnostics Test (May 26th, 2021)					
Parameters				Results	
Color				Yellow	
Appearance				Turbid	
Presence of Coagulum				Negative	
Presence of Blood				Negative	
Total Leukocyte Count (x 10^3^/μL)				21.635 x 10^3^/μL	
Neutrophils (%)				90%	
Lymphocytes (%)				10%	
Monocytes (%)				3%	
Eosinophils (%)				0%	
Red Blood Cells (RBC/HPF)				None	
Glucose (mg/dL)				4 mg/dL	
Lactate Dehydrogenase (μL)				3694 μL	
Total Protein (g/dL)				3.60 g/dL	
Microscopic Features of Fluid (Cytology)				Acute and chronic inflammation comprising several neutrophils and lymphocytes against a proteinaceous background with no malignant cells present.	

Pleural fluid diagnostic results found that the pleural fluid was exudative, with increased neutrophils. No malignant cells were seen on pleural fluid cytology. However, a significant presence of neutrophils and lymphocytes, with high proteinaceous debris in the background, indicated ongoing acute and chronic inflammation. Cultures of the pleural fluid were taken on the same day, with results elaborated in Table 4. A direct acid-fast bacilli (AFB) smear was found to be negative. Cultures of the pleural fluid revealed moderate growth of gram-negative bacilli with pus cells.

**Table 4:** Findings of pleural fluid cultures AFB: acid fast bacilli; ZN: Ziehl-Neelsen

Findings of Pleural Fluid Cultures (May 26th, 2021)			
Gram Stain Test		Results	
Pus Cells		Moderate	
Gram Negative Bacilli		Moderate growth: (1) Heavy growth of *Pseudomonas aeruginosa*; (2) Moderate growth of *Acinetobacter* species	
AFB Direct Smear Test Using (ZN) Stain		Results	
Acid Fast Bacilli		Negative (no growth)	

Dandy-Walker continued to be in the background compared to more acute issues like pneumonia. His pneumonia had exacerbated and led to complications such as sepsis and, eventually, pneumothorax, from which the patient expired. He could not be resuscitated, as his family had signed the do-not-resuscitate code and refused any intervention.

## Discussion

A unique congenital posterior fossa malformation, Dandy-Walker malformation, is diagnosed radiologically by qualities such as fragmented or complete agenesis of the vermis, concerning the cortex and deep cerebellar nuclei, aside from a fourth ventricle having a cyst [10]. The three main classes of the Dandy-Walker complex are Dandy-Walker malformation, Dandy-Walker variant, and Mega Cisterna Magna [1]. It is typically diagnosed at birth and in neonates [14]. Presenting signs of Dandy-Walker malformation in newborns include microcephaly, hypotonia, cleft palate, and cerebellar signs such as convulsions, irritability, and loss of muscle tone and coordination [1]. 

Dandy-Walker malformation is a more severe form than the Dandy-Walker variant. It consists of a cerebrospinal fluid-filled central expansion in the posterior fossa with fourth ventricle dilatation and partial to complete cerebellar agenesis [1,6]. Dandy-Walker is generally found as an incidental finding, with the main investigative choice being MRI [1,6]. This was the case for our patient, who was taken for an MRI after presenting with cerebellar signs such as ataxia and incoordination. As a result, he was found to have Dandy-Walker malformation on radiological grounds. Differentials were reported for the Dandy-Walker variant and Mega Cisterna Magna. Still, the eventual diagnosis was made to be Dandy-Walker malformation after radiological findings, such as dilation of the fourth ventricle, posterior fossa malformation, and hypoplastic vermis, which were classic of the Dandy-Walker malformation type. 

As was the case for our patient, Dandy-Walker can remain undiagnosed until further investigations are done. While our patient presented with cerebellar signs, as seen in the condition, there have been rare reports of other neurologically related issues presented in patients as a result of Dandy-Walker, such as sensorineural hearing loss and ischemic stroke [6,10]. In one study, hydrocephalus and elevated intracranial pressure in adult-onset cases are said to be repeatedly brought about by brainstem and cerebellar deterioration, which could be caused by conditions like Dandy-Walker [15]. Moreover, it can be associated with other conditions, such as trisomy 13 and 18, for example, which can result in further variation in presentations among patients, such as duplicated ureters and ocular findings, with retinal folds being the most common [16,17]. Therefore, it is important to keep an open mind and investigate this, as it presents various signs in each individual differently.

Most research conducted on Dandy-Walker has simply provided general knowledge on the clinical and radiological picture found in neonates, rather than in adults. Regarding case reports, they include childhood presentations of the condition. In contrast, several others discuss presentations in adults - those discussing adult presentations of Dandy-Walker malformation all present unique cases, as previously discussed. In one case, a 75-year-old man with right-sided sensorineural hearing loss and vertigo was referred for an MRI, which found the dilatation of the fourth ventricle, cerebellar hypoplasia, and the absence of the cerebellar vermis. This was a sporadic case, as any linkage of Dandy-Walker to deafness has thus far been undiscovered [5]. Looking at the previous literature, these cases did not present with classic cerebellar signs until adult life and were considered rare or unexpected. These signs in the cases mentioned presented in patients living normal lives until unique signs developed suddenly, thus leading to the respective medical teams investigating the cause, only to find the incidental findings of Dandy-Walker on radiology. This was the same for our patient, who had been living a normal life until cerebellar signs developed late in his adult life. He had lethargy and weakness for two to three years, with no proper investigation being done to rule out the cause, and was only able to have options for treatment close to his expiration.

Although considered the most frequently occurring cerebellar malformation in humans, Dandy-Walker, overall, is a rare case presentation [18]. The Dandy-Walker complex has two major types: notably, the Dandy-Walker malformation and the Dandy-Walker variant. The Dandy-Walker malformation type consists of a cystically enlarged fourth ventricle, an absent or partially present cerebellar vermis, and a dilated posterior fossa [8,18]. The other type, the Dandy-Walker variant, involves cystic dorsal growth with a hypoplastic vermis and a comparatively absent enlargement of the posterior fossa. The condition can occur in association with or as a result of various issues, including genetic anomalies, syndromic malformations, monogenic disorders, and TORCH infections, most commonly rubella, along with syndromes of structural birth defects. To further add, Dandy-Walker malformation has been linked to several types of mutations of certain chromosomes, such as duplication of the 9p gene and deletion of 3q24.3, which contains the ZIC1 and ZIC4 genes, also known as Dandy-Walker malformation genes. Other genes include FOXC1, FGF17, LAMC1, and NID1 [8,18].

There is a lack of new research on the condition, with the same knowledge already known and repeated. While this rare condition is more commonly found in neonates, only 10%-20% are diagnosed late into adolescence or adulthood due to having an asymptomatic presentation [14]. Moreover, Dandy-Walker malformation has a mortality rate of 70% in newborns, the main cause being deformities that occur alongside it [14]. The condition was found to predominantly affect female neonates versus male neonates [6]. However, for cases discussed in adult presentations, Dandy-Walker presented relatively equally in males [5,10,13] and females [11,12]. The reason there may be more information about neonates is due to the frequency and necessity of prenatal imaging. Most neonates present with prominent findings, like hydrocephalus, and show more severe signs and symptoms as early as one year [18]. Dandy-Walker malformation accounts for roughly 7.5% of the incidents of hydrocephalus in infants [18]. Up to half have intellectual or learning disabilities [14], leading to the necessity of medical interventions and early diagnosis.

However, in most cases for adults, these presentations are asymptomatic, leading to missed early diagnosis and treatment until they start presenting with issues later on in life. Moreover, as mentioned, Pakistan lacks imaging equipment [7], which could also lead to missed diagnoses in neonates without prominent issues. This could also be the case for patients with vague symptoms like headaches, unstable gait, facial palsy, muscle spasms, and changes in cognition or behavior, which are commonly found in older children and adults and could be explained by many other differentials before considering Dandy-Walker [14]. Overall, 90% of cases have hydrocephalus [18], a major and life-threatening complication. Of these, 60% of patients with hydrocephalus have motor issues, while 25% have auditory or visual issues [19]. Moreover, 50% of children with hydrocephalus die before age 3, while only 20%-23% of children live adult lives. Of those who reach adulthood, only 38% have a normal mental capacity [19].

Overall, several cases present unique adult presentations of Dandy-Walker malformation. In one case [5], a 75-year-old man was diagnosed after presenting with sensorineural hearing loss, and three years later, he had progressed to having cognitive dysfunction, poor gait, and frequent falls, leading to a state that required full-time nursing care. In another report [13], Dandy-Walker insidiously led to hemorrhagic stroke and eventual death in a patient. These cases did not present with classic cerebellar signs until adult life and were considered rare or unexpected. The signs in these cases presented in patients living normal lives until unique, prominent, and sometimes fatal conditions developed suddenly, leading to the respective medical teams investigating the cause, only to find the incidental findings of Dandy-Walker on radiology [5,13].

In terms of treatments discussed, there are only a few options, such as ventriculoperitoneal shunts for those with severe hydrocephalus and medical management through medication for seizures, diuretics and steroids to lower CSF pressure, and physical therapy [19]. While both options deal with symptomatic care and increasing the quality of life for the patient, there is ultimately no cure. As with any chronic disease, therefore, the options available should be considered, especially physical and speech therapy to help the patients increase their quality of life. Zamora et al. echo the same message that a multidisciplinary team of healthcare workers must be involved to improve the care and outcomes for the patient [18]. Moreover, general physicians and pediatricians should be aware of this issue and be able to comfortably provide a diagnosis and options for medical and surgical treatments. In cases of generally younger patients and female patients of reproductive age, genetic counseling should also be done. The articles echo the same message that this care can only be achieved if healthcare professionals are continuously learning about the condition to diagnose and treat it, and work together to create a detailed plan of management for the patient, maintain ethics, and separate responsibilities according to their field as part of the team and coordinate to provide holistic care.

Moreover, the article explains that neurosurgeons or neurologists should consult the patient in a timely manner. Ventriculoperitoneal shunting has been advised as the surgical treatment of choice, as the risks have proven minimal [8,18]. One study found that it was better to do ventriculoperitoneal shunting on younger children with comorbidities, while children who were older but had fewer comorbidities were better off with endoscopic third ventriculostomy, an alternative option that was mentioned to have similar longevity, low rates of postoperative complications, and the potential for patients to avoid having a permanent shunt [20]. Still, ventriculoperitoneal shunting of hydrocephalus for Dandy-Walker is still not as effective, with increased complications and mortality [19]. A 10-year mortality rate after shunting is 5%-15% in those with non-tumor-related hydrocephalus, but if shunting is done in a timely manner after timely diagnosis, these deaths attributable to this lack would be reduced [19]. Early detection through imaging studies would help patients get the appropriate quality of care and make their symptoms more manageable [18]. The principal imaging modality used for patients is MRI, which is currently the gold standard for diagnosis [8,18].

As a whole, Pakistan is burdened by many third-world diseases, so knowledge of rare diseases like Dandy-Walker is even more lacking. With a population of 147.6 million people in Pakistan, the country has an incredibly limited number of neuro-imaging modalities, with only 80 CT scanners and 19 MRI machines [7]. Moreover, Sajjad explains that in general, while a neuro-radiologist would usually have access to functional MRI and magnetic resonance spectroscopy, this is not present for those in Pakistan [7]. In fact, with such high prices for these scans for patients in a third-world country, these machines, such as MRI machines, only four have diffusion/perfusion imaging, ranging from 2000 to 12000 Rupees. CT scans will cost 5000 Rupees, but they cannot be used for angiography and perfusion CT with an additional 750 for ionic and 1500 for nonionic contrast. This would prevent both the healthcare professionals and the patients from getting a simple scan [7]. Moreover, the care would thus be limited and result in what happened to our patient, where the diagnosis of Dandy-Walker occurred at a hospital that had access to these limited machines. Still, as explained before, quality care comes from wanting the best for the patient, and while these patients could have been referred for scans or transferred much earlier to our hospital, the patient was only referred when he started having a respiratory infection, meaning these blatant signs were not addressed at all previously. The article highlights that image findings can change based on weeks in gestation, and reevaluation must take place at 20 to 22 weeks of pregnancy to confirm the diagnosis [18]. This may be harder as all imaging modalities are very hard to come by in Pakistan, and could only be done in facilities that actually have access to these machines. Overall, timely diagnosis using imaging modalities like MRI and management are key to helping patients with Dandy-Walker [18]. However, the healthcare system in Pakistan is lacking in every aspect.

Moreover, the patient with associated sensorineural hearing loss, who eventually needed nursing care, resembles the state of health that our patient arrived in [5]. This could hint towards the possibility that Dandy-Walker had caused the eventual progression of a bed-bound state. Therefore, it is important to keep an open mind about this condition as a differential and investigate further, as Dandy-Walker may present differently in each individual and can lead to fatal outcomes in some if diagnosis and treatment are delayed until late in life, such as the patient who expired from an associated hemorrhagic stroke. For our patient, Dandy-Walker was also a missed diagnosis until his admission to the General Medicine Department at Dow University Hospital, where the presentation of seeing him in a bed-bound state of extreme lethargy and weakness finally received attention for further investigation. However, it was too late in his life, and his physical state was too weakened to allow for proper treatment. Still, physical therapy and nursing care were advised and started.

While our patient expired due to pneumothorax as a complication of his worsening pneumonia, Dandy-Walker may have been the culprit leading the patient to this deteriorated, bed-bound state and might have led him to aspirate and catch pneumonia in the first place. However, there were many differentials contributing throughout his stay that could have masked the possibility of Dandy-Walker as the direct cause of his altered mental status and deterioration of physical functioning, along with the question as to why the patient was bed-bound. These differentials were bacterial infections leading to pneumonia and cellulitis in the patient, causing an electrolyte shift in the patient. While one could reason that the shift in electrolytes may have caused the patient's deterioration in mental state, it could also be argued that the finding of dilated ventricles and cerebellar hypoplasia, which suggested Dandy-Walker, might have also been the cause of the patient's altered consciousness, lethargy, bed-bound state, and the presence of the cerebellar signs previously mentioned.

Thus, after the results of the MRI, Dandy-Walker became an additional possibility to the causes of the patient's current state, yet the exact knowledge of how severely Dandy-Walker had impacted the patient was hard to tell due to the other simultaneously presenting factors. Moreover, he was admitted for the main issue of suspicion of a respiratory infection, not for neurological issues, showing the gap in the care given in rural areas due to broken health infrastructure in Pakistan. The radiological finding confirmed that the patient presented with Dandy-Walker malformation, a condition that presents commonly in newborns and children but rarely in adults. While our team maintained ethics and discussed physical therapy with the patient’s family, ventriculoperitoneal shunting was not advised due to the patient’s infectious state, age, and frail condition. However, this process of patient management had most likely not been previously carried out in a smaller clinic, where weakness and lethargy can certainly be attributed to old age. Thus, he remained undiagnosed, with no past record of being seen for this condition.

Upon a cerebellar exam, more specific cerebellar signs were observed, and further diagnostic imaging was carried out. This was possible because a hospital admission allows for more time with the patient, along with a more holistic approach to addressing all needs in a patient, whereas a clinic pinpoints what the patient came for. This practice should be changed, and a more holistic approach should be used even in clinics. Moreover, clinics do not have access to the limited imaging machines that hospitals have been provided with, thus taking away focus on conditions that can only be diagnosed through imaging. However, referrals could have still been made to visit a center that had imaging machines. However, again, this patient’s presenting signs of weakness and fatigue were not something that could be alarming for a 76-year-old patient, thus allowing the condition to be overlooked and the diagnosis to be delayed. This situation shows the inconsistent and widespread lack of maintaining the best standards of care for these patients in Pakistan.

While these approaches to maintaining care, as described above, are common in patients with chronic conditions in first-world countries, this is not only a chronic condition but a rare one, with a rare presentation in a 76-year-old male patient in a third-world country like Pakistan, where the health infrastructure is crumbling. With resources lacking and imaging machines only being present in a few healthcare facilities, these resources must be allocated wisely by healthcare professionals. Thus, this could be another major reason why this patient’s rare circumstance also caused him to be overlooked. The economic burden on Pakistan’s healthcare system has made it hard for even patients with the simplest and treatable conditions to be seen, thereby making it even harder for patients like this case to receive attention for all their needs. Overall, the patient presented with relatively unalarming signs over the years and was taken to a clinic where resources and proper care are overlooked, especially in a country like Pakistan. These were all factors that contributed to the delayed diagnosis, and therefore, due to time, there was a lack of treatment being provided to help improve this patient’s outcomes against Dandy-Walker and improve his overall well-being.

## Conclusions

In conclusion, the effects of Dandy-Walker malformation, from one individual to another, can vary significantly. Classically affecting the cerebellum, which plays a chief role in motor and cognitive development, Dandy-Walker malformation can present in those with either normal or abnormal cognition. The 76-year-old male patient had presented with cerebellar signs that were undiagnosed for many years, and only after imaging was it concluded that he had Dandy-Walker malformation, a condition typically seen congenitally. While Dandy-Walker malformation was not the main cause of death, it is likely that the condition played a part in the patient's deteriorating health up to that point.

When cases like these come to light, it is important that they are discussed, as conditions continue to change and present differently with time, and there is a lack of focus in the medical community on rare conditions. Moreover, while it is easier to catch and diagnose neonates, physicians may be more cautious about considering Dandy-Walker as a differential diagnosis and likely will not approach it in such a manner. This patient's health suffered and continued to deteriorate because these issues were not given attention in the past. Rare conditions with no cure must be treated like any chronic condition, with the focus on improving the patient's well-being by diagnosing in a timely manner and exploring all possible routes of care with the patient to prevent deterioration of health and susceptibility to ailments like infections. Advanced imaging modalities can diagnose this disorder. However, such imaging techniques are expensive to implement in developing countries like Pakistan, which also brings us to why such cases of Dandy-Walker malformation can go undiagnosed. More importantly, cases like these provide knowledge that is lacking for rare conditions like Dandy-Walker and call for a change in old textbook knowledge, such as Dandy-Walker being considered only a congenital disease, when it has been shown to also present later on in adulthood. Otherwise, these cases will always be viewed as anything but involving a condition that genuinely affects individuals on a daily basis.
